# A Case Report of Hypoglycemia Due to Late Dumping Syndrome After Jejunostomy Tube Insertion

**DOI:** 10.7759/cureus.6897

**Published:** 2020-02-06

**Authors:** Usamah Elalem, Abdulraof Almahfouz, Abdulrahman Alfadhel, Abdulaziz Almohamedi, Ibrahim Bin Ahmed

**Affiliations:** 1 Internal Medicine, King Faisal Specialist Hospital, Riyadh, SAU; 2 Endocrinology, King Faisal Specialist Hospital, Riyadh, SAU; 3 Emergency Medicine, Alfaisal University, Riyadh, SAU; 4 Psychiatry, Alfaisal University, Riyadh, SAU; 5 Internal Medicine, Alfaisal University, Riyadh, SAU

**Keywords:** jejunostomy, late dumping syndrome, hypoglycemia

## Abstract

Dumping syndrome occurs when food moves to the small bowel faster than usual. Patients can report a variety of symptoms such as abdominal cramps, weakness, flushing, shakiness, and decreased consciousness. Dumping syndrome can be divided into early and late based on the onset of the symptoms after the ingestion of a meal. In the literature, cases of dumping syndrome have been reported, but rarely after jejunostomy tube insertion. We report a case of an 86-year-old female suffering from late dumping syndrome after jejunostomy tube placement. An 86-year-old Saudi female presented with decreased oral intake and gastrostomy tube placement was decided. Later, the multi-disciplinary team agreed to switch her to jejunostomy tube after she developed a couple of complications in the site of insertion. However, she developed hypoglycemia due to late dumping syndrome, which was managed with the addition of starch and switching her back to a gastrostomy tube feeding.

## Introduction

Nowadays, obesity is a global issue affecting 39.8%, according to the 2016 Centers for Disease Control and Prevention (CDC) report. Obesity causes a significant reduction in quality of life. The high prevalence of obesity, in turn, causes an increase in surgical procedures to reduce weight. One of the many complications of such surgical intervention is the dumping syndrome. This phenomenon occurs when food moves too quickly to the small bowel, particularly sugar. According to research, the prevalence of dumping syndrome after a gastric surgical procedure is up to 20%-50%. Severe dumping syndrome up to 1%-5%. Early dumping syndrome occurs more commonly than late dumping syndrome. Early is when symptoms occur within less than an hour after food ingestion. Usually, early dumping syndrome happens due to fluid shifts. However, late dumping syndrome happens due to an exaggerated insulin response to carbohydrates. Also, late dumping syndrome occurs after more than an hour after food consumption [1,2].

There are some cases reported in the literature of dumping syndrome after the insertion of tubes. Also, there are cases reported of dumping syndrome after placing a jejunostomy tube, such as our case. Here, we discuss a case of an 86-year-old female who developed late dumping syndrome after jejunostomy tube insertion [3].

## Case presentation

An 86-year-old female presented to the emergency room with decreased oral intake and agitation that started a few weeks ago with decreased urinary output. No fever or change level of consciousness was noted, but the patient experienced increased agitation. She is a known case of type 2 diabetes mellitus (T2DM) on Janumet (metformin/sitagliptin), with advanced Alzheimer’s disease since 2015, hypertension, dyslipidemia, chronic kidney disease (CKD), status post breast cancer on hormonal therapy (tamoxifen), recurrent urinary tract infection (UTI) requiring multiple hospitalizations, and fracture of the left femur neck that underwent internal fixation in 2014. She has no known allergies to medications or food. Family history was unremarkable. Social history includes a highly supportive family with attendant nurses. Her functional status is described as bed bond, need assistance with daily life activities, poorly communicating, and poor recognition of family members. The patient underwent laboratory workup, suggesting acute kidney injury. She improved after rehydrating her with intravenous (IV) fluids. Swallowing assessment was done, and they noted that she has a delay in the oral phase of swallowing. Most likely due to advanced dementia, the recommendation was to keep her on a puree diet and fluids with thickeners. Dietitian reviewed her case, and after calorie counting, they reported that she has a significant decrease in oral intake. A meeting has been held by a multi-disciplinary team discussing the best alternative method of feeding, keeping in mind patient's co-morbidities. The multi-disciplinary team consisted of family members, a neurologist, dietitian, and a speech therapist. They agreed that the patient is a candidate for alternative feeding with gastrostomy tube insertion, to achieve her daily caloric intake requirements, due to her multiple co-morbidities, advanced dementia, and decreased oral intake.

During the hospital course on January 4, 2018, gastrostomy tube was inserted, and the patient tolerated the procedure with no complications. Furthermore, feeding was started the next day, when the nurse noted swelling around the gastrostomy tube site, for that reason, feeding was stopped, and IV fluids were initiated. On January 6, 2018, the patient started to spike fever and develop further swelling around the gastrostomy tube insertion site. Ultrasound was done, which shows no signs of any collections, hematoma, or edema. Also, broad-spectrum antibiotics were started after sending the septic workup. On January 7, 2018, the patient continued to spike high-grade fever, increase agitation, and started to become hypotensive. Her gastrostomy tube site showed redness, induration, and her abdomen was tender. She was given IV fluids, and antibiotics were upgraded. Computed tomography (CT) of the abdomen was done and showed a misplaced gastric tube in the upper abdominal wall at the level of the gastric antrum and free peritoneal gas suggestive of a leak, but no extensive drainable collections.

**Figure 1:**
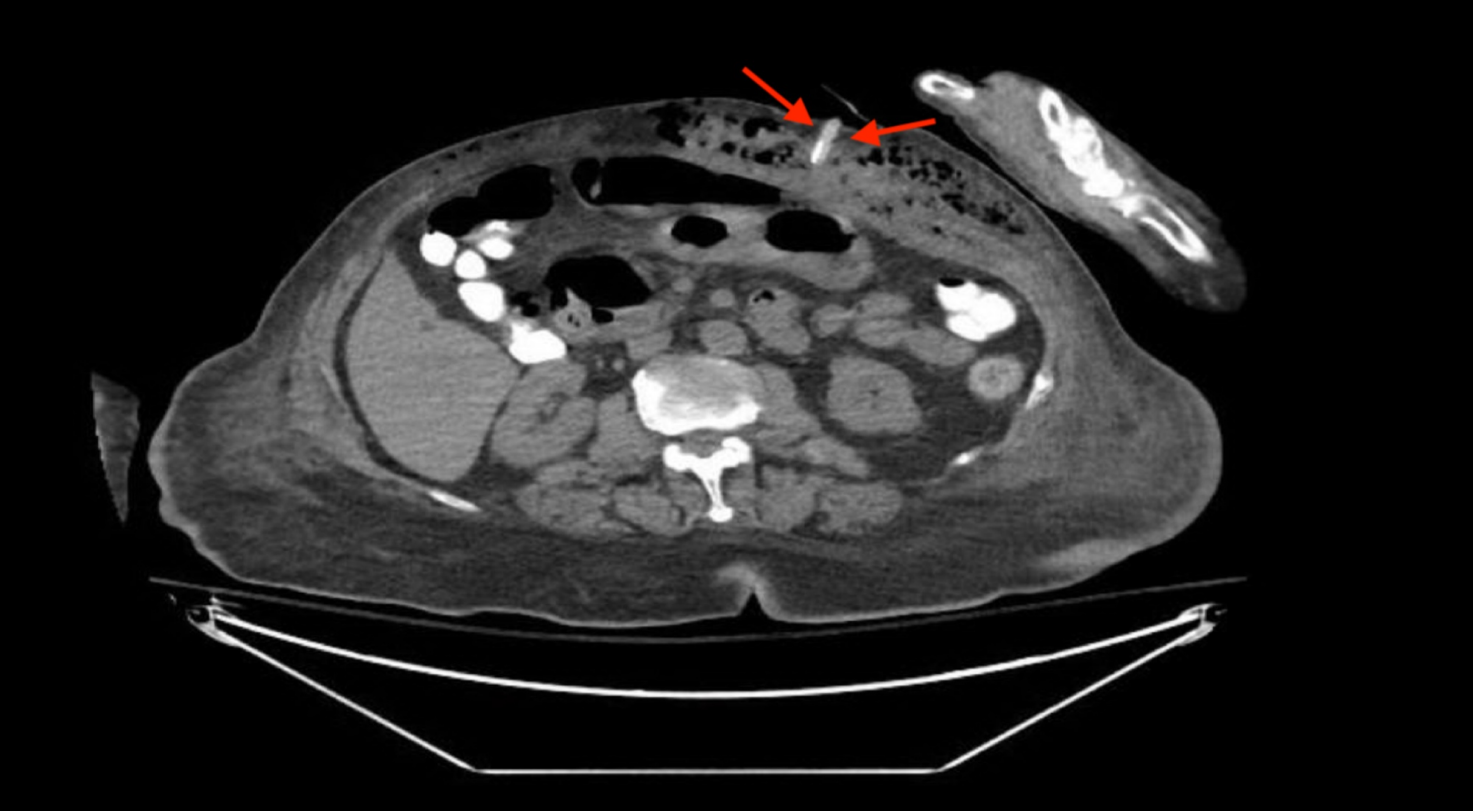
Computed tomography (CT) of the abdomen which shows the misplaced gastric tube in the upper abdominal wall at the level of the gastric antrum

The surgical team was consulted, and she was taken to the operating room and underwent closure of the gastrostomy tube site with debridement along with jejunostomy tube insertion. Her inflammatory markers improved, and feeding was started. On June 28, 2018, the patient noted to have low blood sugar during a routine checkup, 3.6 mmol/dl, with no apparent symptoms of hypoglycemia. Her oral hypoglycemic agents were stopped six months earlier. Over the last few months, she had normal blood sugar, and her last HbA1c was 5.4% in April 2018. Moreover, her weight reduced from 85 kg to 62 kg. Although the patient has no signs of sepsis, and was on continuous jejunostomy tube feeding 60 ml/hr and on continuous dextrose 10% 50 ml/hr, her blood glucose remained low. All differentials were ruled out, and they increased feeding to 70 ml/hr. The dietitian changed her formula to a low carbohydrate diet with glucerna, but still with no improvement. However, after adding starch to her feeding, the patient’s blood sugar normalized within 48 hours, and dextrose infusion was stopped.

On June 30, 2018, the patient complained of lower limb swelling with no other symptoms. Dietitian increased protein and albumin supplementation to 1.5 mg/kg. Nevertheless, the albumin level remained low with generalized anasarca. The case was discussed with the surgical team regarding changing the jejunostomy tube to gastrostomy tube as it will improve hypoglycemia and low albumin. After changing the tube and resuming feeding, the patient blood sugar and albumin improved. 

## Discussion

In general, when patients cannot tolerate oral feeding, tube feeding is used to provide the required caloric intake. Dumping syndrome can be early or late. When symptoms occur in less than an hour, the term early dumping syndrome is used. It is usually caused by fluid shifts. Symptoms of early dumping syndrome include pain in the abdomen, nausea, and diarrhea. When symptoms occur after more than an hour, the term late dumping syndrome is used. It is characterized by changes in the fluid influx or the release of hormones. Symptoms of late dumping syndrome include feeling hungry, loss of consciousness, and sweating [4].

However, different differential diagnoses must be taken into consideration before considering dumping syndrome, such as adrenal insufficiency, sepsis, insulinoma, drug-induced, or malignancy. 

Obesity is a strong indication for bariatric surgeries. Furthermore, as obesity rates are high, weight loss surgeries are increasing. One of the many complications of weight-reducing surgeries is dumping syndrome. The occurrence of dumping syndrome after bariatric surgery is more common in Laparoscopic Roux-en-Y gastric bypass than in laparoscopic sleeve gastrectomy [4].

Late dumping syndrome after jejunostomy tube insertion is rare, but some cases have been reported in the literature. A case of a 70-year-old woman was reported in 2016, for suffering from postprandial hypoglycemia due to late dumping syndrome after direct feeding with jejunostomy tube. They reported the resolution of the postprandial hypoglycemia caused by the late dumping syndrome by using miglitol and a formula containing isomaltulose. Management is by enteral feeding with low glycemic indexes. Moreover, it usually involves different specialties to tackle it adequately [5].

## Conclusions

In conclusion, late dumping syndrome after jejunostomy tube is rare. However, we report a case of an 86-year-old female complaining of hypoglycemia happening because of late dumping syndrome after jejunostomy tube feeding. The patient's blood sugar improved with the addition of starch. The patient albumin level improved after changing her jejunostomy tube to a deep gastrostomy tube. Nevertheless, different differentials such as adrenal insufficient, sepsis, malignancies, or drug-induced should be ruled out first. 
